# Learning from real world data about combinatorial treatment selection for COVID-19

**DOI:** 10.3389/frai.2023.1123285

**Published:** 2023-04-03

**Authors:** Song Zhai, Zhiwei Zhang, Jiayu Liao, Xinping Cui

**Affiliations:** ^1^Biostatistics and Research Decision Sciences, Merck & Co., Inc., Rahway, NJ, United States; ^2^Department of Statistics, University of California, Riverside, Riverside, CA, United States; ^3^Biostatistics Innovation Group, Gilead Sciences, Foster City, CA, United States; ^4^Department of Bioengineering, University of California, Riverside, Riverside, CA, United States

**Keywords:** G-computation, virtual multiple matching, subgroup analysis, multiple comparisons with the best, COVID-19

## Abstract

COVID-19 is an unprecedented global pandemic with a serious negative impact on virtually every part of the world. Although much progress has been made in preventing and treating the disease, much remains to be learned about how best to treat the disease while considering patient and disease characteristics. This paper reports a case study of combinatorial treatment selection for COVID-19 based on real-world data from a large hospital in Southern China. In this observational study, 417 confirmed COVID-19 patients were treated with various combinations of drugs and followed for four weeks after discharge (or until death). Treatment failure is defined as death during hospitalization or recurrence of COVID-19 within four weeks of discharge. Using a virtual multiple matching method to adjust for confounding, we estimate and compare the failure rates of different combinatorial treatments, both in the whole study population and in subpopulations defined by baseline characteristics. Our analysis reveals that treatment effects are substantial and heterogeneous, and that the optimal combinatorial treatment may depend on baseline age, systolic blood pressure, and c-reactive protein level. Using these three variables to stratify the study population leads to a stratified treatment strategy that involves several different combinations of drugs (for patients in different strata). Our findings are exploratory and require further validation.

## 1. Introduction

COVID-19, a respiratory illness caused by the coronavirus SARS-CoV-2, is an unprecedented global pandemic that is adversely affecting virtually every part of the world (Helmy et al., 2020). As of February 22, 2023, the global number of confirmed cases has risen above 674 million, with over 6 million COVID-19-related deaths reported worldwide (overall mortality rate ~1.02%), according to the Johns Hopkins University Coronavirus Resource Center (https://coronavirus.jhu.edu). The increasing availability of COVID-19 vaccines is hugely helpful in the fight against COVID-19. That being said, given the magnitude of the pandemic, searching for effective treatments of COVID-19 will likely remain an important scientific question in the foreseeable future. The United States Food and Drug Administration has approved the antiviral agent remdesivir for treating adults and certain pediatric patients and has authorized several monoclonal antibody treatments for emergency use. The approval of remdesivir was based on the results of three randomized clinical trials, including a double-blinded, placebo-controlled trial in which remdesivir significantly reduced the median time to recovery from 15 to 10 days (Beigel et al., 2020). In November 2021, Pfizer announced that a new antiviral pill, Paxlovid, was showing promising results in clinical trials, where Paxlovid significantly reduced the proportion of people with COVID-19 related hospitalization or death by 88% compared to placebo (https://www.fda.gov/news-events). But because the data of our study was collected in the early of pandemic (2020) when Paxlovid was not available, the recurrence of COVID-19 treatment with Paxlovid had not been reported. Other therapeutic agents, such as hydroxychloroquine, have also been studied, often with negative or inconclusive results (Wu et al., 2020). There is also growing interest in combining multiple agents to improve efficacy (e.g., Hung et al., 2020). Bassetti et al. (2021) give a summary of the available clinical evidence about the efficacy and safety of various antiviral agents for treating COVID-19. While COVID-19 is much better understood now than it was when it first broke out, much remains to be learned about how best to treat the disease while considering patient and disease characteristics.

This paper reports a case study of combinatorial treatment selection for COVID-19 based on real-world data from early days of the pandemic. The data were collected on a cohort of 417 consecutive COVID-19 patients admitted to the Second Affiliated Hospital of Southern University of Science and Technology in Shenzhen, China, between January 11, 2020 and February 16, 2020. Their disease status was confirmed using reverse transcription-polymerase chain reaction (RT-PCR). Baseline information was collected on age, sex, body mass index, disease severity, comorbidities, imaging features, and 49 biochemical variables (e.g., oxygen saturation); some of these are summarized in Table 1. The patients had an average age of 45 years (SD 17.7) and were largely evenly distributed between the two sexes (47.5% male). Most patients (74.1%) had moderate disease, and some (17.5%) had severe disease, with relatively few (3.8% and 4.6%, respectively) in the mild and critical disease categories.

**Table 1:** Summary of demographics and baseline characteristics for all COVID-19 patients (measured at initial admission to hospital), treatment success group and treatment failure group.

**Characteristic**	**Total, *N* = 417**	**Treatment success, *N* = 321**	**Treatment failure, *N* = 96**
**Age, years**			
Mean (SD)	45.2 (17.7)	46.8 (17.1)	40.1 (18.7)
0–30 years, *n* (%)	75 (18.0)	46 (14.3)	29 (30.2)
31–60 years, *n* (%)	242 (58.0)	190 (59.2)	52 (54.2)
61+ years, n (%)	100 (24.0)	85 (26.5)	15 (15.6)
**Sex**, ***n*** **(%)**			
Male	198 (47.5)	159 (49.5)	39 (40.6)
**BMI, kg/m2, mean (SD)**	23.1 (3.64)	23.2 (3.61)	22.7 (3.72)
**Severity classification**, ***n*** **(%)**			
Mild	16 (3.8)	13 (4)	3 (3)
Moderate	309 (74.1)	234 (73)	75 (78)
Severe	73 (17.5)	59 (18)	14 (14.6)
Critical	19 (4.6)	15 (5)	4 (4.2)
**Comorbidity**, ***n*** **(%)**			
Hypertension	86 (20.6)	68 (21.2)	18 (18.8)
Diabetes mellitus	31 (7.4)	23 (7.2)	8 (8.3)
Coronary heart disease	25 (6.0)	22 (5.3)	3 (3.1)
Active cancer	6 (1.4)	5 (1.6)	1 (1.0)
Chronic obstructive pulmonary disease	15 (3.6)	12 (3.7)	3 (3.1)
Hepatitis B	13 (3.1)	10 (3.1)	3 (3.1)
**Imaging feature**, ***n*** **(%)**			
Lung consolidation	92 (22.1)	62 (19.3)	30 (31.2)
Ground-glass opacity	367 (88.0)	282 (87.9)	85 (88.5)
Pulmonary infiltration	325 (77.9)	247 (76.9)	78 (81.3)
Pleural effusion	17 (4.1)	11 (3.4)	6 (6.3)

Data shown are *n* (%) or mean (SD).

While hospitalized, the patients were treated for COVID-19 using a variety of drugs: five antiviral drugs (lopinavir-ritonavir-arbidol, interferon, oseltamivir, ribavirin, and favipiravir), two anti-inflammatory drugs (methylprednisolone and tocilizumab), and the immunomodulator hydroxychloroquine. As shown in Table 2A, the most commonly used (per patient) drugs were interferon (83.2%), lopinavir-ritonavir-arbidol (79.9%) and methylprednisolone (24.5%). These drugs were frequently combined, so the total percentage in Table 2A is well above 100%. For example, a majority of patients (64%) were treated with interferon and lopinavir-ritonavir-arbidol upon admission; depending on patient conditions and physician judgement, the initial regimen may continue unchanged or be modified in some way. With little guidance available for treating COVID-19, drug choices tended to be exploratory and haphazard. We define a combinatorial treatment as the collection of all drugs administered to a patient during hospitalization, regardless of the actual dosing and timing of specific drugs. Table 2B shows the most common combinatorial treatments adopted for this cohort. Some treatments in Table 2B represent unique combinations of drugs, while others (123+ and “the rest”) are aggregates of different combinations that are difficult to study separately due to low frequencies. Supplementary Figure S1 depicts and compares the eight treatment groups with respect to five baseline covariates (selected in Section 3.4).

**Table 2:** Summary of individual drugs **(A)** and combinatorial drugs **(B)** adopted for the study cohort, together with related outcome information.

**A**.				
**Individual drug**, ***n*** **(%)**	**Code**	**Total**, ***N*** **= 417**	**Failure cases**, ***N*** **= 96**	**Failure rate**
Lopinavir-ritonavir-arbidol	**1**	333 (80)	71 (74)	0.213
Interferon	**2**	347 (83)	80 (83)	0.231
Methylprednisolone	**3**	102 (24)	22 (23)	0.216
Tocilizumab	**4**	7 (2)	2 (2)	0.286
Oseltamivir	**5**	65 (16)	11 (12)	0.169
Ribavirin	**6**	82 (20)	16 (17)	0.195
Favipiravir	**7**	11 (3)	6 (6)	0.545
Hydroxychloroquine	**8**	26 (6)	9 (9)	0.346
**B**.				
**Combinatorial drugs**, ***n*** **(%)**		**Total**, ***N*** **= 417**	**Failure cases**, ***N*** **= 96**	**Failure rate**
**1**		31 (8)	9 (9)	0.290
**2**		34 (8)	9 (9)	0.265
**12**		116 (28)	28 (29)	0.241
**123**		46 (11)	7 (7)	0.152
**125**		31 (7)	5 (5)	0.161
**126**		34 (8)	6 (6)	0.176
**123+** ^a^		39 (9)	9 (9)	0.231
**The rest** ^b^		86 (21)	23 (24)	0.267

a Lopinavir-ritonavir-arbidol-interferon-methylprednisolone with one or more drugs.

b The rest: all the other possible combinations.

The outcome of interest in this case study is treatment failure, defined as death during hospitalization or recurrence of COVID-19 within four weeks of discharge. Recurrence after discharge is not just an individual health issue but also has a potential impact on public health. Post-discharge death without recurrent COVID-19 is considered a competing risk in this definition. While hospitalized, patients were tested every other day. Two consecutive negative test results were required for discharge. All discharged patients were subject to strict quarantine for four weeks, either at home or at a designated location. Follow-up visits were performed every 3–5 days during the quarantine. In the end, there were only three deaths in the study cohort, all of which occurred during hospitalization. Ninety three of the 414 discharged patients were found to have recurrent COVID-19 during the quarantine. Thus, a total of 96 patients in the study cohort experienced treatment failure (primarily due to recurrence), with an overall failure rate of 23%.

The overarching objective of this case study is to estimate and compare the failure rates of the eight combinatorial drug treatments in Table 2B, both in the whole study population and in subpopulations defined by baseline characteristics. Such comparisons will shed light on the relative efficacy of treatments and help identify the most promising treatments for further investigation. A major analytical challenge is the likely presence of confounding in this observational study, where treatment assignment was not randomized. For example, patients in treatment groups 123 and 123+ tended to be older than patients in other treatment groups (see Supplementary Figure S1). Due to possible confounding, the observed failure rates in Table 2B may be biased as estimates of population-level failure rates. Additional challenges include (relatively) high-dimensional covariates and their complex relationship with treatment outcomes, which will be addressed using modern machine learning (ML) methods. ML methods have been employed in many research areas related to COVID-19, such as medical imaging, disease diagnosis, vaccine development, and drug design (Asada et al., 2021; Dong et al., 2021; Perez Santin et al., 2021; Xu et al., 2022). The present article adds to this literature with a new application (i.e., drug effectiveness analysis, especially multi-drug combination analysis).

The rest of the article is organized as follows. In Section 2.1, we formulate the problem, state key assumptions, and give a rationale for choosing the G-computation approach (Robins, 1986) over various propensity score methods for confounding adjustment. In Section 2.2, we describe specific methods, including a virtual multiple matching (VMM) method for estimating covariate-specific failure rates, a synthetic minority over-sampling technique (SMOTE) for improving the performance of random forest with class-unbalanced data, a permutation test for the sharp null hypothesis of no treatment effect on any patient, and a multiple comparisons with the best (MCB) procedure for demonstrating the superiority of one treatment to all the other treatments. The main results of our analysis are presented in Section 3, followed by a discussion in Section 4 and conclusion remarks in Section 5. Additional technical details and results are provided as online Supplementary material.

## 2. Methodology

### 2.1. General framework

We start by formulating research questions using potential outcomes (Rubin, 1974). Let T denote the set of combinatorial drug treatments under consideration (see Table 2B). For each t∈T, let *Y*(*t*) be the potential outcome (1 for failure; 0 for success) that would result if a patient receives treatment *t*. Denote by T∈T the actual treatment received and *Y* = *Y*(*T*) the actual outcome observed (assuming consistency and stable unit treatment value). Let X∈X be a vector of baseline (i.e., pre-treatment) covariates that may be associated with the potential outcomes for one or more treatments; these include the variables in Table 1 as well as some biochemical variables. The observed data based on *n* = 417 subjects will be conceptualized as independent copies of (*X, T, Y*) and denoted by (*X*_*i*_, *T*_*i*_, *Y*_*i*_), *i* = 1, …, *n*.

As indicated earlier, our case study will address the following research questions:

1. To test the sharp null hypothesis of Fisher (1935), which in the present setting states that *Y*(*t*) does not depend on *t* for any patient in the study population. Formally, this may be written as *Y*(*t*_1_) ≡ *Y*(*t*_2_) for any pair of treatments (*t*_1_, *t*_2_). Successful rejection of the sharp null hypothesis would indicate that a non-null treatment effect exists, at least for some patients.
2. To estimate and compare the overall failure rates of the different treatments, defined as π_*t*_ = P{*Y*(*t*) = 1} for t∈T. Such a comparison will help identify the overall best treatment: $t_{\text{opt}}={arg min}_{t\in T}\pi_{t}$.
3. To estimate and compare the failure rates of the different treatments in selected subpopulations, defined as π_*t*_(*A*) = P{*Y*(*t*) = 1|*X* ∈ *A*} for t∈T and A⊂X with P(*X* ∈ *A*) > 0 (measure-theoretic issues are ignored throughout). Such a comparison will help identify the best treatment for patients in the chosen subpopulation: $t_{\text{opt}}\left(A\right)={arg min}_{t\in T}\pi_{t}\left(A\right)$.

To deal with possible confounding in an observational study, we assume that all important confounders are included in *X* so that treatment assignment is ignorable (Rosenbaum and Rubin, 1983) upon conditioning on *X*:


(1) $$\begin{array}{l} P\left\{Y\left(t\right)=1|X,T=t\right\}=P\left\{Y\left(t\right)=1|X\right\}=:p_{t}\left(X\right),\;t\in T. \end{array}$$

This assumption implies that, in each subpopulation defined by *X*, the study is practically a randomized experiment in the sense that treatment assignment is independent of potential outcomes (Rosenbaum, 1984; Robins, 1986). We also assume that no patient is *a priori* excluded from receiving any treatment in T:


(2) $$\begin{array}{l} P\left(T=t|X\right)>0,\;t\in T. \end{array}$$

This is commonly known as the positivity assumption. The practical implication of this assumption is that there should be sufficient overlapping between the covariate distributions in different treatment groups (Imbens, 2004). Assumptions 1 and 2 together ensure that the functions *p*_*t*_, t∈T, are nonparametrically identified as


(3) $$\begin{array}{l} p_{t}\left(X\right)=P\left(Y=1|X,T=t\right). \end{array}$$

This further implies the nonparametric identifiability of


(4) $$\begin{array}{l} \pi_{t}\left(A\right)=E\left\{p_{t}\left(X\right)|X\in A\right\} \end{array}$$

for all t∈T and suitable A⊂X. In particular, $\pi_{t}=\pi_{t}\left(X\right)$ is identified as E{*p*_*t*_(*X*)} for all t∈T.

Numerous statistical methods are available for confounding adjustment under the assumptions stated above. These include the G-computation (or outcome regression) approach (Robins, 1986), which in the present setting amounts to estimating *p*_*t*_(*X*) from Eq. 3 and substituting the estimate into Eq. 4. There are many alternative methods that involve the propensity score (Rosenbaum and Rubin, 1983), defined originally for binary treatments and generalized later to multi-level treatments (Imbens, 2000). An estimated propensity score can be used for stratification (Rosenbaum and Rubin, 1984), matching (Rosenbaum and Rubin, 1985), weighting (Robins, Hernan and Brumback, 2000), or constructing doubly robust, locally efficient estimators (e.g., Van Der Laan and Robins, 2003; Tsiatis, 2006; Van Der Laan and Rose, 2011). These propensity score methods can in principle be applied or adapted to our case study; however, their implementation is not straightforward with multiple treatments under consideration. Furthermore, when a propensity score method is used for subgroup analysis, it may be difficult to ensure compatibility of estimates across subgroups. It is not clear, for instance, that a propensity score method will necessarily respect the relationship


(5) $$\begin{array}{l} \pi_{t}=P\left(X\in A\right)\pi_{t}\left(A\right)+P\left(X\notin A\right)\pi_{t}\left(X\backslash A\right), \end{array}$$

and a severe violation of this relationship would make the results difficult to interpret. The G-computation approach does not have such difficulties with interpretation and is straightforward to implement. Therefore, the G-computation approach will be employed in our case study to answer all of our research questions in a unified fashion.

### 2.2. Specific methods

**Figure 1:**
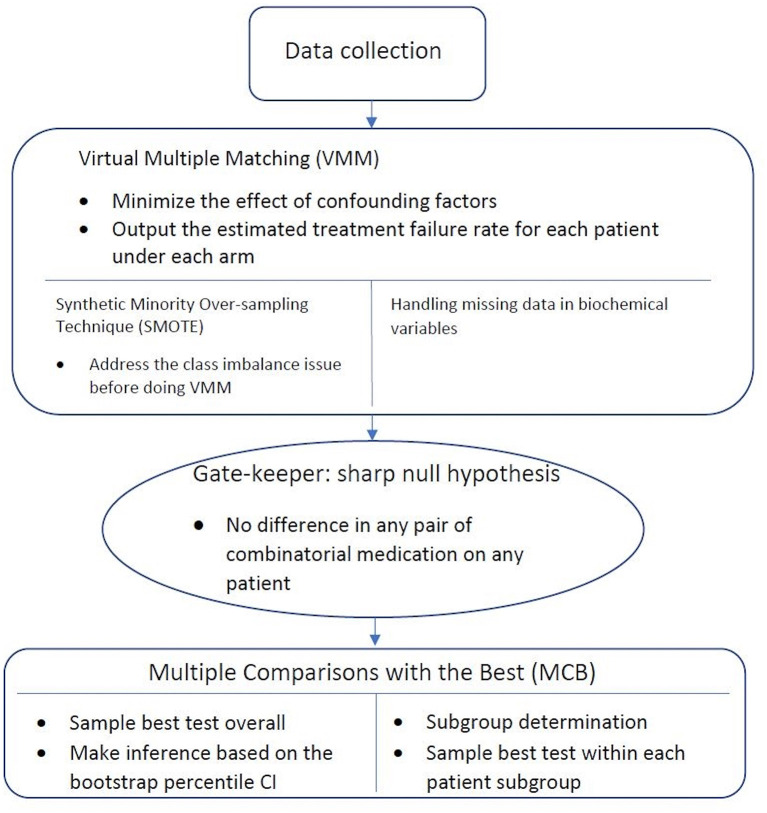
Workflow of statistical analysis. After the data is collected, Virtual Multiple Matching (VMM) is first performed to minimize the effect of confounding factors. SMOTE is applied with the class imbalance, and missing indicator method is applied with missing values. Sharp null hypothesis is then conducted as the gate-keeper to test if there is an overall combinatorial treatment efficacy. Finally, Multiple Comparisons with the Best (MCB) is performed to select the best drug combination strategy within each patient subgroups, stratified in a data-driven manner.

Figure 1 gives an overview of the workflow of our statistical analysis, with specific methods described below.

#### 2.2.1. Virtual multiple matching

Under the G-computation approach, we work with the pseudo-observations $R_{it}={\hat{p}}_{t}\left(X_{i}\right)$, *i* ∈ {1, …, *n*}, t∈T, where ${\hat{p}}_{t}$ is an estimate of *p*_*t*_ obtained by regressing *Y*_*i*_ on *X*_*i*_ among subjects with *T*_*i*_ = *t*, as suggested by equation 3. For treatment evaluation and comparison, each *R*_*it*_ will be used as a surrogate outcome for subject *i* under treatment *t*. This is somewhat similar to the “virtual twins” approach of Foster et al. (2011) for subgroup selection in a randomized clinical trial. Our setting is an observational study, and we use the surrogate outcomes *R*_*it*_ to adjust for possible confounding under the assumptions stated in Section 2.1. We will refer to this approach as virtual multiple matching (VMM) to acknowledge the fact that multiple treatments are currently under consideration. In Foster et al. (2011), where *T* is binary, the random forest algorithm is used to regress *Y* on (*T, X, T* × *X*). The use of random forest seems appropriate for our case study with dozens of baseline covariates in *X*, for it would be extremely difficult to specify an approximately correct logistic regression model for predicting *Y* on the basis of (*X, T*). On the other hand, even for a binary *T*, it has been noted that regressing *Y* on (*T, X, T* × *X*) may provide limited flexibility for accommodating different outcome-covariate relationships in different treatment groups (Lu et al., 2018). For maximal flexibility with eight treatments under consideration, we grow a separate random forest for each treatment t∈T to estimate *p*_*t*_ and obtain *R*_*it*_, *i* = 1, …, *n*. Section 3.1 provides empirical evidence supporting this approach. The random forest estimation is carried out using the randomForest package in R with the default configuration except ntree = 1000 and nodesize = 3.

#### 2.2.2. Synthetic minority over-sampling technique

In the language of machine learning, treatment failure is the minority class in our case study, with observed failure rates 15–29% in the eight treatment groups. The presence of class imbalance is known to cause performance issues with classification algorithms such as random forest (Ali, Shamsuddin and Ralescu, 2015; Brownlee, 2019), and a synthetic minority over-sampling technique (SMOTE) is available to improve classification performance on class-imbalanced data (Chawla et al., 2002). Even though our estimation problem is not a classification problem, we will consider SMOTE as a possible way to improve estimation performance. The idea is to augment the data (i.e., subjects with *T*_*i*_ = *t*) with artificial instances of the minority class (i.e., treatment failures) that resemble the existing ones, so that the augmented dataset has a lesser imbalance issue. Clearly, the addition of artificial treatment failures may create an upward bias in the estimation of *p*_*t*_ and thus requires statistical adjustment. Details on the implementation of SMOTE as well as the subsequent adjustment in our case study are provided in Supplementary Method. Empirical evidence supporting the use of SMOTE is given in Section 3.1.

#### 2.2.3. Handling of missing data

Most of the 49 biochemical variables in *X* have some missing values, with a mean (rsp. median) proportion of 34% (rsp. 31%) missing values across variables. (There are no missing values in (*Y, T*) or the other components of *X*.) Excluding subjects with missing values in *X* would incur a substantial loss of information and could potentially result in biased estimates. We include all available participants in the analysis using a missing indicator method (Miettinen, 1985; Greenland and Finkle, 1995; Burton and Altman, 2004; Donders et al., 2006). Specifically, we include all subjects in the estimation procedure by treating missing values as a special category. Each covariate with missing values is represented by two derived variables: an indicator for missingness (1 if missing; 0 if observed) and an “imputed” version of the original variable with missing values replaced by an arbitrary value, say 0. It is easy to see that the two derived variables together carry exactly the same information contained in the original (partially observed) variable.

#### 2.2.4. Permutation test of sharp null hypothesis

The aforementioned techniques will be used to obtain $R_{it}={\hat{p}}_{t}\left(X_{i}\right)$ from {(*X*_*i*_, *Y*_*i*_):*T*_*i*_ = *t*} for all (*i, t*). Once computed, the *R*_*it*_'s will be used to test the sharp null hypothesis stated in Section 2.1. This null hypothesis implies that *Y*_*i*_ = *Y*_*i*_(*t*) for all (*i, t*), regardless of the actual treatments *T*_*i*_. Therefore, permuting the treatment labels {*T*_*i*_, *i* = 1, …, *n*} randomly provides a valid reference distribution for any test statistic. If the sharp null hypothesis is false, *Y*_*i*_(*t*) will depend on *t* and we expect larger differences in $\left\{R_{it},t\in T\right\}$, at least for some *i*. To capture such differences, our test statistic is defined as a sum of within-patient variances:


$$\overset{n}{\underset{i=1}{\sum }}\frac{1}{|T|-1}\underset{t\in T}{\sum }{\left(R_{it}-R_{i\cdot }\right)}^{2},$$

where |T| is the size of the set T and $R_{i\cdot }=|T{|}^{-1}\underset{t\in T}{\sum }R_{it}$. This test statistic will be computed for both the original sample (to find the observed value) and a large number of permuted samples (to produce a reference distribution). The proportion of values in the permutation-based reference distribution that exceed the actual observed value will be used as the *p*-value for testing the sharp null hypothesis.

#### 2.2.5. Estimation of failure rates

For each treatment t∈T, the overall failure rate π_*t*_ is estimated as ${\hat{\pi }}_{t}=n^{-1}\overset{n}{\underset{i=1}{\sum }}R_{it}$, and the subpopulation failure rate π_*t*_(*A*) is estimated as


$${\hat{\pi }}_{t}\left(A\right)=\overset{n}{\underset{i=1}{\sum }}I\left(X_{i}\in A\right)R_{it}/\overset{n}{\underset{i=1}{\sum }}I\left(X_{i}\in A\right),$$

where *I*(·) is the indicator function, for any subpopulation *A* with sufficient representation in the data (i.e., $\overset{n}{\underset{i=1}{\sum }}I\left(X_{i}\in A\right)$ is not too small). Confidence intervals for these quantities will be obtained using a nonparametric bootstrap percentile method. Each bootstrap sample will be obtained by sampling from the original dataset {(*X*_*i*_, *T*_*i*_, *Y*_*i*_), *i* = 1, …, *n*} with replacement. The resulting bootstrap sample is $\left\{\left(X_{i},T_{i},Y_{i}\right),i\in I\right\}$, where I consists of *n* i.i.d. observations from the uniform distribution on {1, …, *n*}. Each bootstrap sample will be analyzed using the exact same methods for obtaining ${\hat{\pi }}_{t}$ and ${\hat{\pi }}_{t}\left(A\right)$ for all *t* and *A* of interest.

#### 2.2.6. Multiple comparisons with the best

In comparing ${\hat{\pi }}_{t}$ across *t*, a natural question is whether the apparent winner, say ${\hat{t}}_{\text{opt}}={\mathrm{argmin}}_{t}{\hat{\pi }}_{t}$, is significantly better than the other treatments in the sense of having a lower failure rate. This question can be addressed using a multiple comparisons with the best (MCB) procedure (Hsu, 1984; Cui et al., 2021). The null hypothesis being tested is that ${\hat{t}}_{\text{opt}}$ is not strictly better than the other treatments, that is,


$$\pi_{{\hat{t}}_{\text{opt}}}-\underset{t\neq {\hat{t}}_{\text{opt}}}{\min}\pi_{t}\geq 0.$$

To test this hypothesis at level α, one may use the same nonparametric bootstrap procedure described earlier to obtain a 1−α upper confidence bound for


$${\hat{\pi }}_{{\hat{t}}_{\text{opt}}}-\underset{t\neq {\hat{t}}_{\text{opt}}}{\min}{\hat{\pi }}_{t}$$

The null hypothesis will be rejected if the upper confidence bound is less than 0. This MCB procedure can also be performed in a subpopulation *A* after replacing π_*t*_ with π_*t*_(*A*) and ${\hat{\pi }}_{t}$ with ${\hat{\pi }}_{t}\left(A\right)$.

#### 2.2.7. Subgroup selection

The preceding discussion of subgroup analysis is for a given subgroup of interest. In our case study, subgroups are not pre-specified but will be chosen in a data-driven manner. Specifically, we use the random forest algorithm to assess the importance of each baseline variable for predicting the average failure rate of all treatments under consideration. This will identify a small number of variables with the highest (overall) prognostic values, which may or may not be effect modifiers. (An effect modifier must be prognostic for one or more treatments, but a prognostic variable may have no effect modification at all.) The strongest prognostic variables identified in this manner will be examined further as potential effect modifiers. A variable is considered a possible effect modifier if it stratifies the study cohort into subgroups with different treatment choices (i.e., estimated best treatments). Once identified, the apparent effect modifiers will be considered jointly in developing a stratified treatment strategy.

## 3. Data analysis results

### 3.1. Validation of VMM and SMOTE

**Figure 2:**
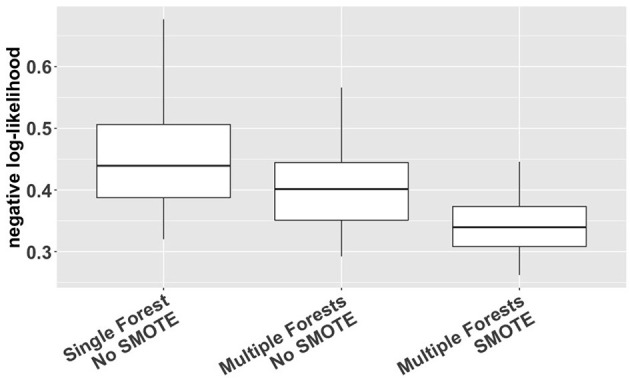
Cross-validated negative log-likelihood for three different versions of VMM (single forest vs. multiple forests; with or without SMOTE) based on 1,000 replicates of five-fold cross-validation.

We used a cross-validation approach with the negative log-likelihood as a loss function (Van Der Laan and Dudoit, 2003; Buja et al., 2005) to compare different options of VMM (single forest vs. multiple forests; with or without SMOTE). The log-likelihood value of a regression model is a way to measure the goodness of fit for a model. The lower the value of the negative log-likelihood, the better a model fits a dataset. The comparison was based on 1000 simulation replicates of random partitioning. Within each replicate, a standard five-fold cross-validation procedure was performed to compute the cross-validated negative log-likelihood for different estimation methods. The 1000 replicates differed from each other in how the study subjects were actually partitioned into five folds. The results, shown in Figure 2, indicate that VMM with multiple forests fits better than VMM with a single forest (i.e., negative log-likelihood 0.40 vs. 0.44) and that adding SMOTE to VMM with multiple forests produces even better fits than the multi-forest based VMM without SMOTE (i.e., negative log-likelihood 0.34 vs. 0.40). Therefore, VMM with multiple forests and SMOTE was chosen as our estimation method in the rest of the analysis. Using the same cross-validation process, we also compared different choices of ntree (100, 500, 1000 and 2000), and the results suggest that the behavior of our chosen VMM estimation method is not sensitive to ntree (see Supplementary Table S1).

**Figure 3:**
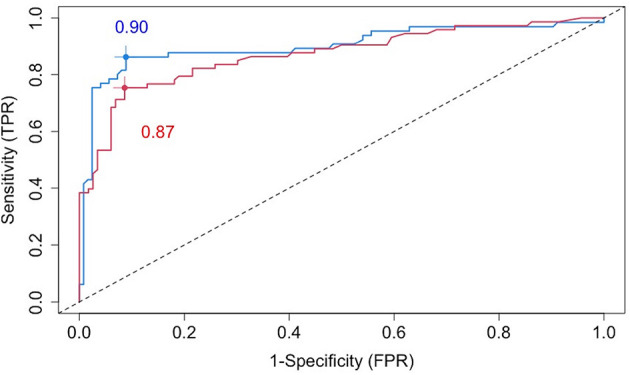
Cross-validated ROC curves for estimated failure probabilities based on all baseline covariates (blue line) or the five most prognostic variables (i.e., the top five variables ranked by the variable importance from random forests): age, SBP, AST, BMI, and CRP (red line).

The blue line in Figure 3 is a cross-validated receiver operating characteristic (ROC) curve for the chosen VMM method based on all baseline covariates (including age, sex, BMI, disease severity, comorbidities, imaging features, and 49 biochemical variables). The area under the curve (AUC) is estimated to be 0.90, demonstrating high prediction accuracy of our proposed VMM algorithm.

### 3.2. Test of sharp null hypothesis

The permutation test of the sharp null hypothesis produced a p-value of 0.008 (see Supplementary Figure S2). This is strong indication that, at least for some patients, differences exist between the failure rates of the eight combinatorial drugs. This result strengthened the motivation for comparing the different treatments in the whole population as well as selected subpopulations.

### 3.3. Overall comparison of treatments

**Figure 4:**
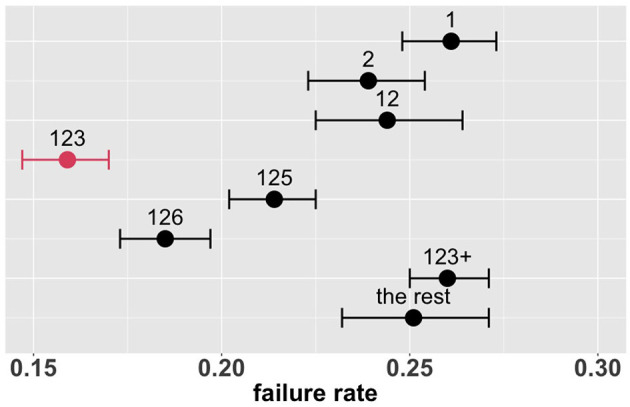
Estimated overall failure rates and 95% confidence intervals for the eight combinatorial drug treatments under consideration (with no adjustment for multiplicity) (1: lopinavir-ritonavir-arbidol, 2: interferon, 3: methylprednisolone, 4: tocilizumab, 5: oseltamivir, 6: ribavirin, 7: favipiravir, 8: hydroxychloroquine).

Figure 4 shows the estimated overall failure rates of the eight combinatorial drug treatments together with 95% confidence intervals. Based on these results, the combination 123 appears to be the most promising treatment for the entire patient population, with an estimated overall failure rate of 16.6%. Supplementary Figure S3 shows the MCB test results with parameters of interest formulated as $\pi_{k}-\underset{i\neq k}{\min}\pi_{i}$, where π denotes the failure rate and *k* denotes the combinatorial drug treatment group specified in Table 2: *k* ∈ {1, 2, 12, 123, 125, 126, 123+, the rest}. In Supplementary Figure S3, the upper bound of the 95% bootstrap percentile confidence interval of $\pi_{123}-\underset{i\neq 123}{\min}\pi_{i}$ is less than zero, indicating that the drug combination 123 is significantly better than the other treatments with the lowest failure rate at the one-sided 2.5% level.

This overall comparison provides some evidence in favor of the combination 123 (if a single treatment is to be chosen for the entire patient population). However, treatment effects may be heterogeneous, and the optimal treatment may depend on patient and disease characteristics. This possibility is addressed in the following subgroup analysis.

### 3.4. Subgroup analysis

In this section, we aim to identify important subgroups of patients, compare treatments in each subgroup, and develop a stratified treatment strategy.

**Figure 5:**
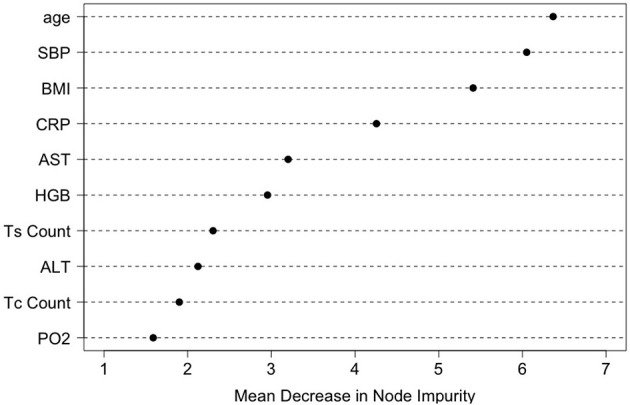
Variable importance ranking by the random forests in terms of the total decrease in node impurity (i.e., residual sum of squares) from splitting on the variable, averaged over all trees.

To identify a small number of covariates for patient stratification, we regressed all *R*_*it*_'s together on *X*_*i*_ using the random forest algorithm and assessed variable importance based on node impurity. Specifically, for each variable, the sum of the Gini impurity decrease across every tree of the forest is accumulated every time that variable is chosen to split a node. The sum is divided by the number of trees in the forest to yield an average. The ten most important variables (i.e., with the largest mean decrease in node impurity) are shown in Figure 5. In our subgroup analysis, we consider subgroups defined using the top five covariates in Figure 5: age, systolic blood pressure (SBP), body mass index (BMI), c-reactive protein (CRP), and aspartate aminotransferase (AST). These five covariates together account for most of the predictive power of *X*, as Figure 3 shows that the ROC curve based only on the five covariates has an AUC of 0.87 (close to the AUC for the ROC curve based on all covariates, i.e., 0.90). Separately from our work, these covariates have been found to be predictive of COVID-19 severity and outcomes (e.g., Ali, 2020; Mahase, 2020; Wei et al., 2020; Zhou et al., 2020; Zuin et al., 2020).

In order to define subgroups, the top five covariates were discretized as follows. SBP was dichotomized at 120 mm Hg; that is, an SBP reading below 120 mm Hg is considered normal and a reading of 120 mm Hg or more is considered high (Robinson and Brucer, 1939). AST was dichotomized at 40 U/L; that is, the normal range for AST is from 5 to 40 U/L and an AST level above 40 U/L is considered high and indicative of liver damage (https://www.medicinenet.com/liver_blood_tests). Following CDC guidelines, we considered patients with 18.5 < BMI < 24.9 as normal, and those with BMI > 30.0 as obese. According to Mayo Clinic, a CRP level greater than 10 mg/L is a sign of severe infection or inflammation. Different cutoffs for age have been suggested in the literature. In our case study, age was trichotimized at 30 and 60 years, as suggested by CDC (cdc.gov/coronavirus) and Li et al. (2020).

**Figure 6:**
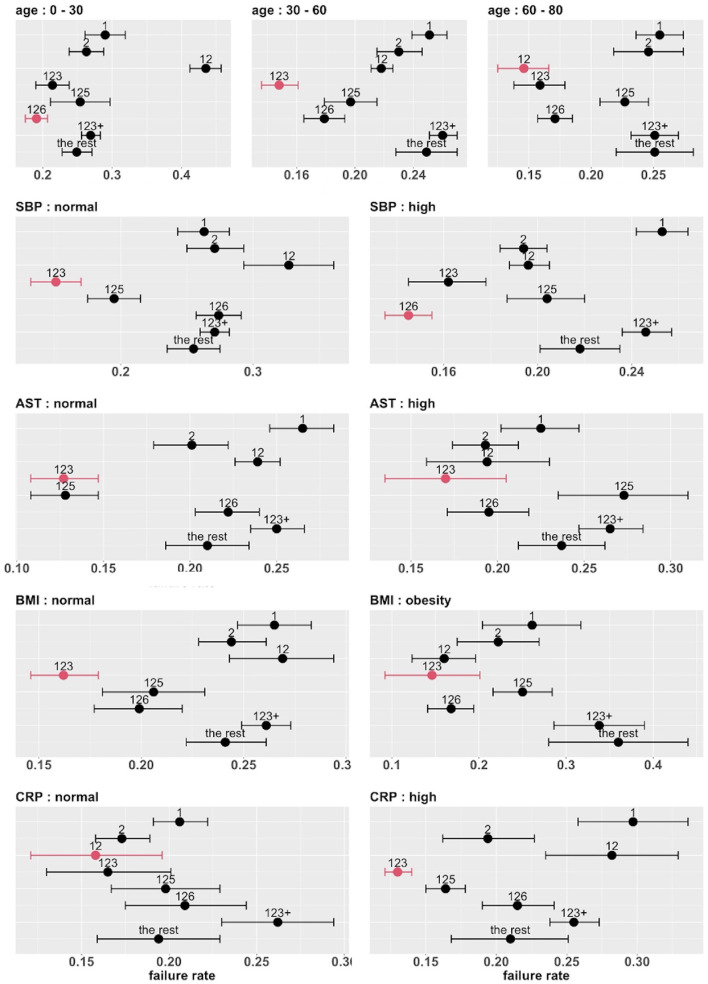
Estimated failure rates and 95% confidence intervals for the eight combinatorial drug treatments under consideration (with no adjustment for multiplicity), in subgroups defined by the top five covariates (one at a time). The lowest estimated failure rate in each subgroup is shown in red.

The top five covariates, which were initially selected on the basis of their overall prognostic value, may or may not be important effect modifiers. Before these covariates were combined for stratification, each covariate was evaluated separately as a potential effect modifier. For each covariate, we estimated and compared the failure rates of the eight combinatorial treatments in each subgroup defined by the covariate. If the estimated optimal treatment (i.e., the treatment with the lowest estimated failure rate) differed across subgroups defined by the same covariate, the covariate was then considered potentially useful for treatment selection. Figure 6 shows the main results (point estimates and 95% confidence intervals) of this evaluation. The corresponding MCB test results are provided in Supplementary Figure S4. The combinatorial treatment 126 had the lowest observed failure rate among young patients (<30). For patients 30–60 years old, drug combination 123 was superior to the other treatments with upper bound of MCB confidence interval less than zero. For patients older than 60, drug combination 12 outperformed the other treatments with the lowest observed failure rate. We therefore considered the age as a potential effect modifier. Similarly, we selected SBP and CRP as potential effect modifiers for a stratified treatment strategy. Meanwhile, BMI and AST were dropped since the optimal combinatorial treatment remained unchanged across different levels of BMI and AST. We summarized our observations from Figure 6 and Supplementary Figure S4 into Table 3.

**Table 3:** Selecting effect modifiers among age, SBP, BMI, CRP, and AST.

**Covariate**	**Subgroup**	**Optimal drug combination**	**Effect modifier**	**Covariate**	**Subgroup**	**Optimal drug combination**	**Effect modifier**
SBP	Normal	123	Yes	BMI	Normal	123	No
	High	126			Obesity	123	
Age	0 - 30	126	Yes	CRP	Normal	12	Yes
	30 - 60	123			High	123	
	60 - 80	12		AST	Normal	123	No
	High	123					

The combinatorial treatment with the lowest observed failure rate varies across different patient subgroups stratified by the effect modifier.

**Figure 7:**
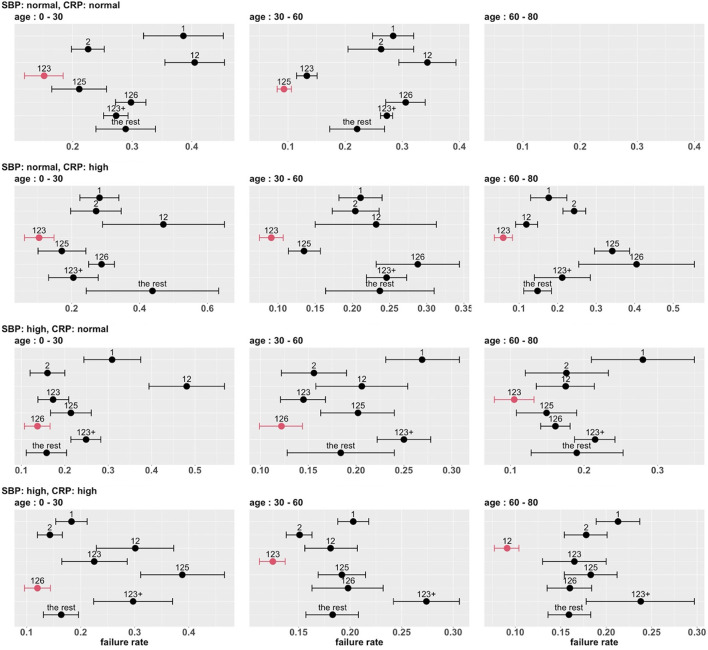
Estimated failure rates and 95% confidence intervals for the eight combinatorial drug treatments under consideration (with no adjustment for multiplicity), in 3 × 2 × 2 subgroups defined by age, SBP and CRP. The lowest estimated failure rate in each subgroup is shown in red.

To develop a stratified treatment strategy, we divided the whole cohort into 3 × 2 × 2 = 12 subgroups by intersecting the previously defined subgroups based on age, SBP and CRP. One of these subgroups (age 60 – 80 with normal SBP and normal CRP) had no data and thus could not be studied. In each of the other 11 subgroups, we estimated and compared the failure rates of the eight combinatorial drug treatments. The point estimates and 95% confidence intervals are shown in Figure 7, and the corresponding MCB test results are presented in Supplementary Figure S5. Table 4 summarizes our main findings (i.e., the best combinatorial treatment selection in different patient subgroups) based on both point estimate results (from Figure 7) and MCB test results (from Supplementary Figure S5), and displays the most promising treatment (with the lowest estimated failure rate) in each of the 11 subgroups studied. In five subgroups (shown as red in Table 4), the apparent optimal treatment is significantly better than all other treatments at the one-sided 2.5% level according to the MCB test. The other subgroups tend to be smaller and have less data. The double combination of lopinavir-ritonavir-arbidol with interferon (12) is included in all the patient subgroups, either used alone or combined with other drugs. The triple combination of lopinavir-ritonavir-arbidol, interferon, and methylprednisolone (123) is the most frequently suggested treatment in Table 4, consistent with the overall comparison in Section 3.3. However, Table 4 also suggests two other treatments (i.e., triple combinations of lopinavir-ritonavir-arbidol, interferon with either oseltamivir (125) or ribavirin (126)) for specific subgroups; these would have been missed without a subgroup analysis.

**Table 4:** A stratified treatment strategy based on age, SBP and CRP.

**SBP**	**CRP**	**Age**		
		0 - 30	30 - 60	60 - 80
Normal	Normal	123	125	
Normal	High	123	123	123
High	Normal	126	126	123
High	High	126	123	12

The combinatorial treatment strategy, that appears to be the best with the lowest observed failure rate (i.e., point estimate), varies across different patient subgroups. The red color indicates statistical significance at the one-sided 2.5% level from the MCB test.

## 4. Discussion

Combinatorial treatment selection for COVID-19 is one of the most pressing issues in today's medical research. In this study, we provide a standard analysis framework for the drug combination problem: (1) apply virtual multiple matching to adjust for possible confounding across multiple treatment groups (SMOTE is applied with the presence of class imbalance); (2) apply missing indicator method to handle missing values in the data; (3) conduct permutation test of overall treatment efficacy; (4) determine patient stratification with identified effect modifiers in a data-driven manner; (5) perform multiple comparisons with the best to select the best drug combination strategy within each subgroup.

In addition, the retrospective case study in this paper also provides some insights on this issue based on real-world data from China. Our case study indicates that, among the treatments adopted in the study cohort, combining lopinavir-ritonavir-arbidol with interferon and possibly other drugs is generally a promising treatment strategy. On one hand, taking lopinavir-ritonavir-arbidol (antiviral drug), interferon (antiviral drug), and methylprednisolone (anti-inflammatory drug) leads to the best outcome overall. On the other hand, there appears to be some treatment effect heterogeneity, and the optimal combinatorial treatment may depend on baseline age, SBP, and CRP. Using the three baseline variables together to stratify the study population leads to a stratified treatment strategy that involves several combinations of drugs (for different strata) with varying levels of evidence. Specifically, for young patients (<30), taking lopinavir-ritonavir-arbidol and interferon, together with methylprednisolone or ribavirin (antiviral drug), leads to the lowest treatment failure rate. However, our MCB test results also indicate that there are other potential drug combinations providing comparable treatment outcomes. For patients older than 30, there exists some particular drug combination that is significantly superior to other combinatorial strategies in most of patient subgroups stratified by SBP and CRP.

The results of the case study should be interpreted cautiously with several limitations in mind. First, the study is limited to a biologically homogeneous patient population in China, and to a relatively small set of drugs (excluding, for example, remdesivir and Paxlovid). Second, conducted in the early days of the pandemic, the study is unable to account for subsequent genetic variations of the SARS-CoV-2 virus. Third, the analysis is retrospective and the selection of subgroups is somewhat *ad hoc* (although the prognosis-based selection of variables and the data-independent choices of cutpoints should provide some protection against possible selection bias). Fourth, the moderate sample size does not allow us to study treatment effects at a very detailed level (i.e., considering doses and times of drug usage). Fifth, a longitudinal causal analysis is infeasible due to lack of information on time-dependent confounders (i.e., daily evaluations of patient conditions). In light of these limitations, our findings should be regarded as exploratory and hypothesis-generating.

In this article, treatment selection is based solely on efficacy. Guo et al. (2021) propose a statistical method to utilize the Food and Drug Administration's adverse event reporting system to compare and select existing drugs for COVID-19 treatment based on their safety profiles. In future research, it will be of interest to combine efficacy and safety in COVID-19 treatment selection.

It should be noted that the methodology used in this case study is fairly general and readily applicable to another observational study of COVID-19 or another disease condition. The code to implement the methodology is available at https://github.com/zhaiso1/COVID19. In this era of big data, there are many disease registries available, which typically measure hundreds of variables on thousands of patients. Such disease registries offer great opportunities to learn about treatment selection from real world data using sophisticated machine learning methods.

## 5. Conclusion

In this retrospective case study of combinatorial treatment selection for COVID-19, we investigate eight different treatment combination strategies with eight candidate drugs. Our contributions are at least three-fold. First, we build a standard analysis pipeline for the drug combination problem, involving a series of novel/popular machine learning and multiple testing algorithms (i.e., VMM, SMOTE, missing indicator method, variable importance ranking, and MCB). The code to implement the analytical pipeline is available at Github https://github.com/zhaiso1/COVID19. Second, our findings emphasize the benefit of combination therapy with correctly combined drugs, compared to the monotone therapy. Third, our case study provides an insight that different patient subgroups may react differently to the same combinatorial drug treatment, depending on patients' demographics and drug mechanisms (e.g., antiviral, anti-inflammatory, immunomodulator). In a nutshell, we believe our study can be used as a foundation for combination therapy of COVID-19, and our analysis framework can be used by stakeholders in public health to address the similar problems in various of disease fields.

## Data availability statement

The raw data supporting the conclusions of this article will be made available by the authors, without undue reservation.

## Ethics statement

Ethical review and approval was not required for the study on human participants in accordance with the local legislation and institutional requirements. Written informed consent from the participants' legal guardian/next of kin was not required to participate in this study in accordance with the national legislation and the institutional requirements.

## Author contributions

JL collected the data. JL and XC designed the study. SZ, ZZ, JL, and XC analyzed the data and contributed to the writing of the manuscript. All authors contributed to the article and approved the submitted version.
