# Supplementary material for: Learning from real world data about combinatorial treatment selection for COVID-19
Source: Front Artif Intell. 2023 Apr 3;6:1123285. doi: 10.3389/frai.2023.1123285 (PMC10106735; doi:10.3389/frai.2023.1123285)
Supplement: Supplementary file 1 [file Data_Sheet_1.pdf]

Supplementary Materials to "Learning from Real World Data about Combinatorial Treatment Selection for COVID-19"

Song Zhai[1,2], **Zhiwei Zhang**[3] **Jiayu Liao***[4] **and Xinping Cui***[2]

[1]*Biostatistics and Research Decision Sciences, Merck & Co., Inc., Rahway, NJ, United States, e-mail:* song.zhai@merck.com

[2]*Department of Statistics, University of California, Riverside, Riverside, CA, United States, e-mail:* song.zhai@merck.com, xinping.cui@ucr.edu

[3]*Biostatistics Innovation Group, Gilead Sciences, Foster City, CA, United States, e-mail:* zhiwei_zhang@yahoo.com

[4]*Department of Bioengineering, University of California, Riverside, Riverside, CA, United States, e-mail:* jliao@engr.ucr.edu

Supplementary Method: Technical Details of SMOTE

Because the random forest algorithm is to be applied to each treatment group separately, we will implicitly focus on a generic treatment group in describing the SMOTE procedure. Let x_0 be the value of X for a subject who experienced treatment failure (i.e., $Y = 1$). Instead of replicating the value x_0, it is computationally advantageous to create new values randomly in a (relatively) small neighborhood of x_0 (Ali, Shamsuddin and Ralescu, 2015; Brownlee, 2019). Specifically, for specified positive integers c and k with $c \leq k$, let $x_1, \dots, x_c$ be chosen randomly from the k nearest neighbors of x_0 among the observed values of X. Let $u_1, \dots, u_c$ be generated randomly and independently from the uniform distribution on the unit interval. The SMOTE procedure creates c synthetic subjects with covariate values

$$u_j x_0 + (1 - u_j) x_j, \qquad j = 1, \dots, c.$$

These synthetic subjects are considered treatment failures in applying the random forest algorithm.

The SMOTE procedure entails a statistical adjustment, which can be derived as follows. Let f_y denote the conditional density of X given $Y = y \in \{0, 1\}$ with respect to a common measure. By Bayes' law, we have

$$\frac{\mathrm{P}(Y = 1|X)}{\mathrm{P}(Y = 0|X)} = \frac{\mathrm{P}(Y = 1) f_1(X)}{\mathrm{P}(Y = 0) f_0(X)},$$

so that

$$\mathrm{logit}\{\mathrm{P}(Y = 1|X)\} = \mathrm{logit}\{\mathrm{P}(Y = 1)\} + \log\{r(X)\},$$

where $r(X) = f_1(X)/f_0(X)$ is the density ratio. Consider a deterministic SMOTE procedure in which each failure ($Y = 1$) is replicated c times into c failures with the same covariate value X. This process changes the marginal odds of $Y = 1$ vs $Y = 0$ by a multiplicative factor of c, without changing the conditional distribution of X given Y. Using P^* to denote probability after SMOTE, we then have

$$\frac{\mathrm{P}^*(Y = 1)}{\mathrm{P}^*(Y = 0)} = c \times \frac{\mathrm{P}(Y = 1)}{\mathrm{P}(Y = 0)}$$

and

$$\begin{aligned} \mathrm{logit}\{\mathrm{P}^*(Y = 1|X)\} &= \mathrm{logit}\{\mathrm{P}^*(Y = 1)\} + \log\{r(X)\} \\ &= \log(c) + \mathrm{logit}\{\mathrm{P}(Y = 1)\} + \log\{r(X)\} \\ &= \log(c) + \mathrm{logit}\{\mathrm{P}(Y = 1|X)\}. \end{aligned}$$

It follows that

$$\mathrm{P}(Y = 1|X) = \mathrm{expit}\{\mathrm{logit}\{\mathrm{P}^*(Y = 1|X)\} - \log(c)\},$$

where expit is the inverse of the logit function. The last equation allows us to adjust an estimate of $\mathrm{P}^*(Y = 1|X)$ (obtained after SMOTE) into an estimate of $\mathrm{P}(Y = 1|X)$, which is of interest to us. Derived for a deterministic SMOTE procedure, this adjustment can be used as an approximation for a random SMOTE procedure with small perturbations in X.

Supplementary Figures

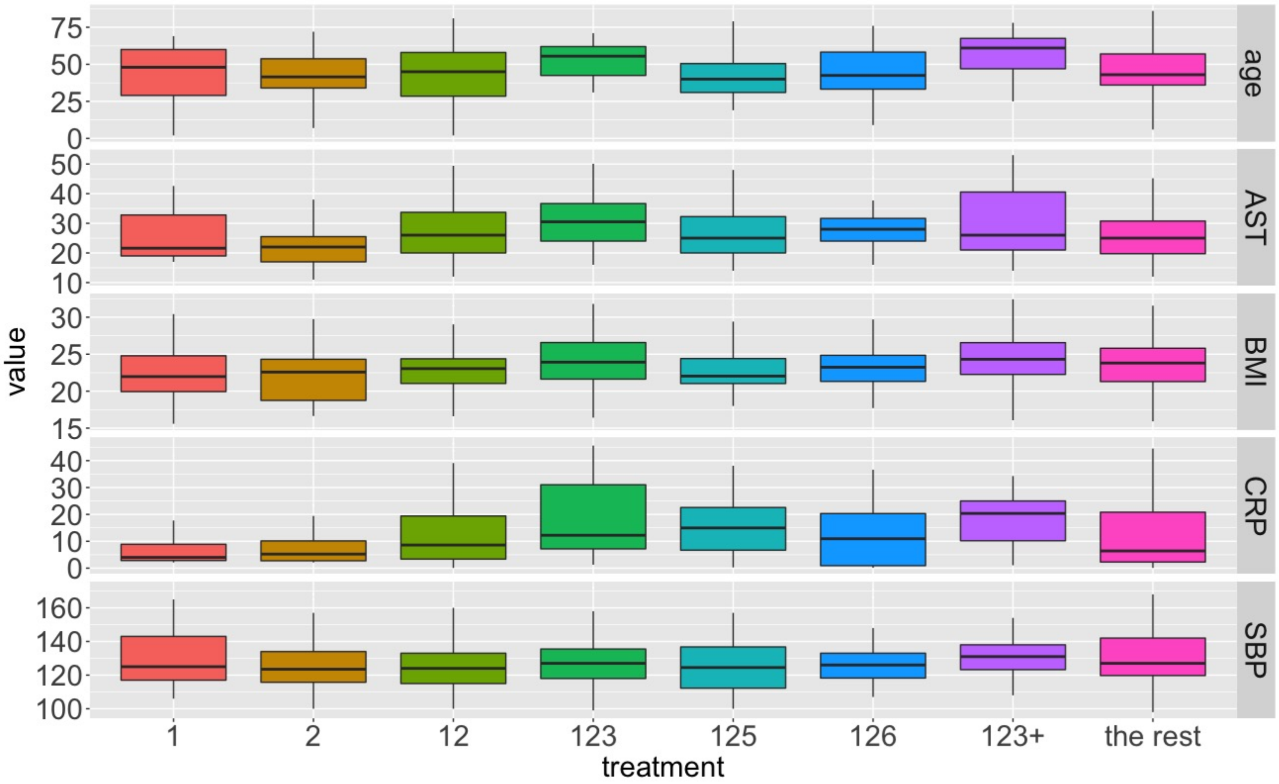

Figure S1: Empirical distributions of age, aspartate aminotransferase (AST), body mass index (BMI), c-reactive protein (CRP) and systolic blood pressure (SBP) in eight combinatorial treatment groups.

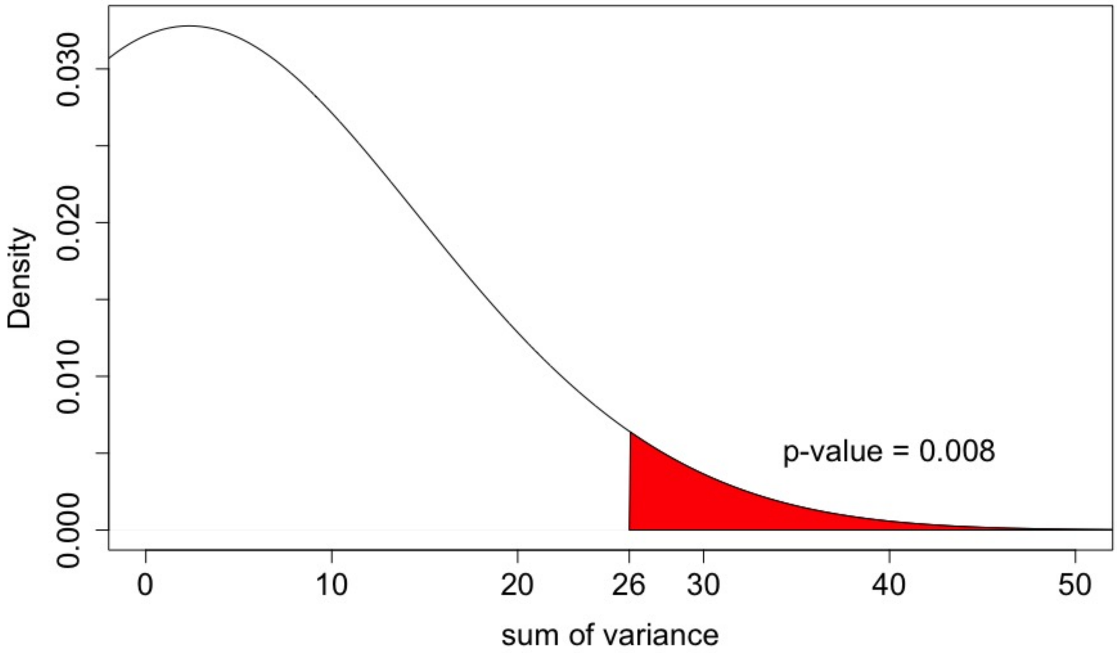

Figure S2: Permutation test of the sharp null hypothesis of no treatment difference for any patient. The observed value of the test statistic is approximately 26, with a p-value of 0.008.

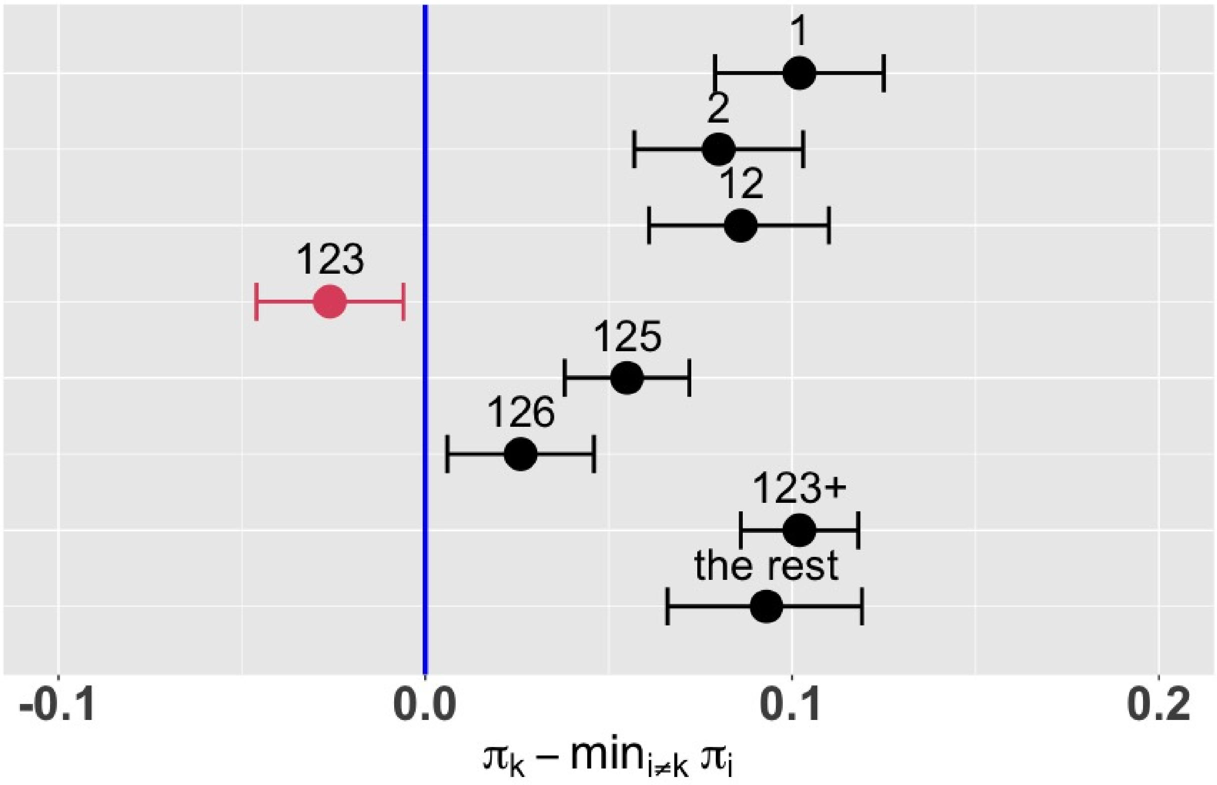

Figure S3: MCB sample best test for drug combination 123 (red) based on a bootstrap percentile 95% confidence interval for $\pi_{123} - \min_{t \neq 123} \pi_t$. The upper confidence bound being less than 0 indicates that the combination 123 is significantly better than the other treatments. Also shown (in black) are MCB bootstrap percentile confidence intervals for the other treatments.

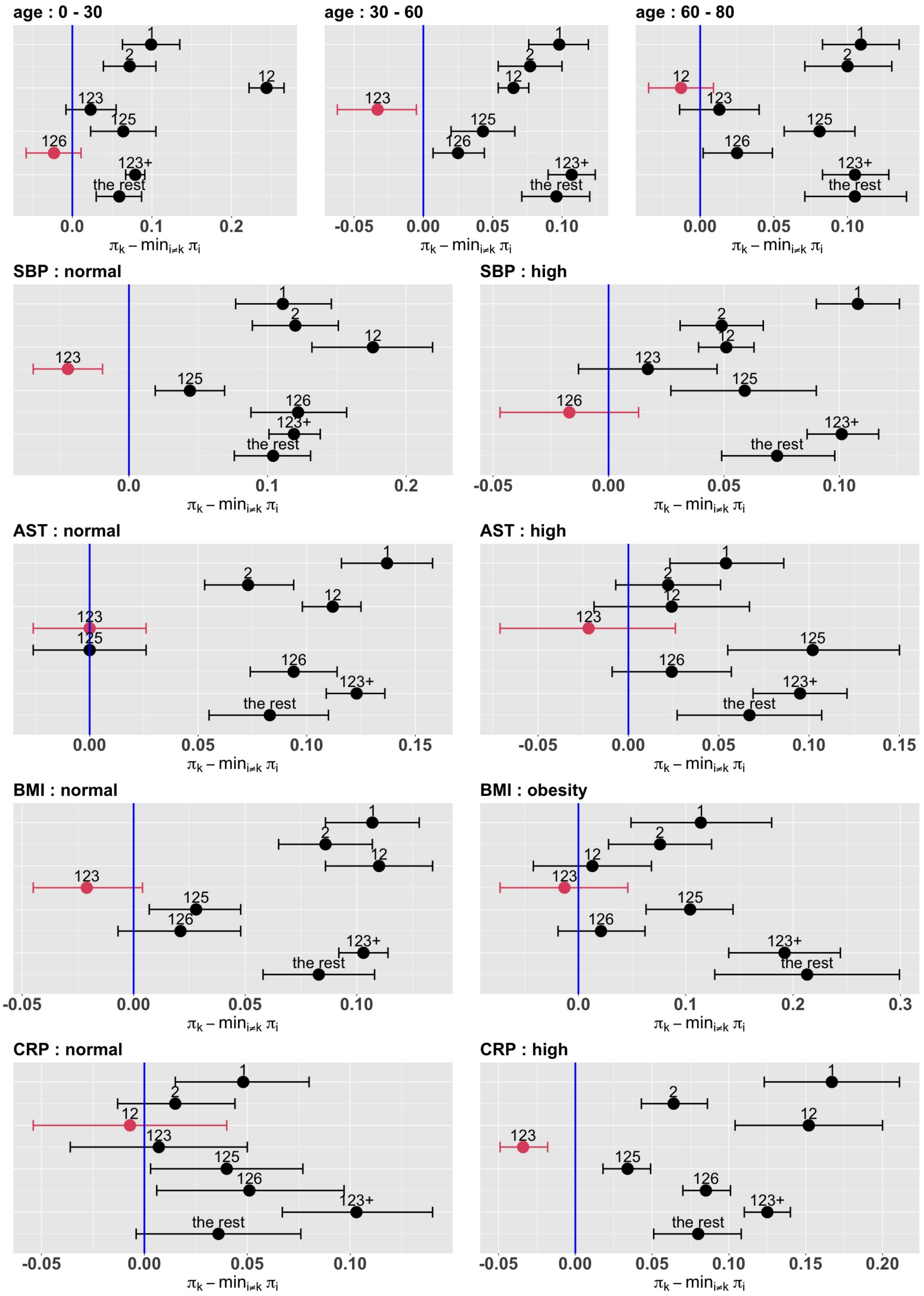

Figure S4: MCB sample best test (red) within each stratum defined by age, SBP, AST, BMI, and CRP.

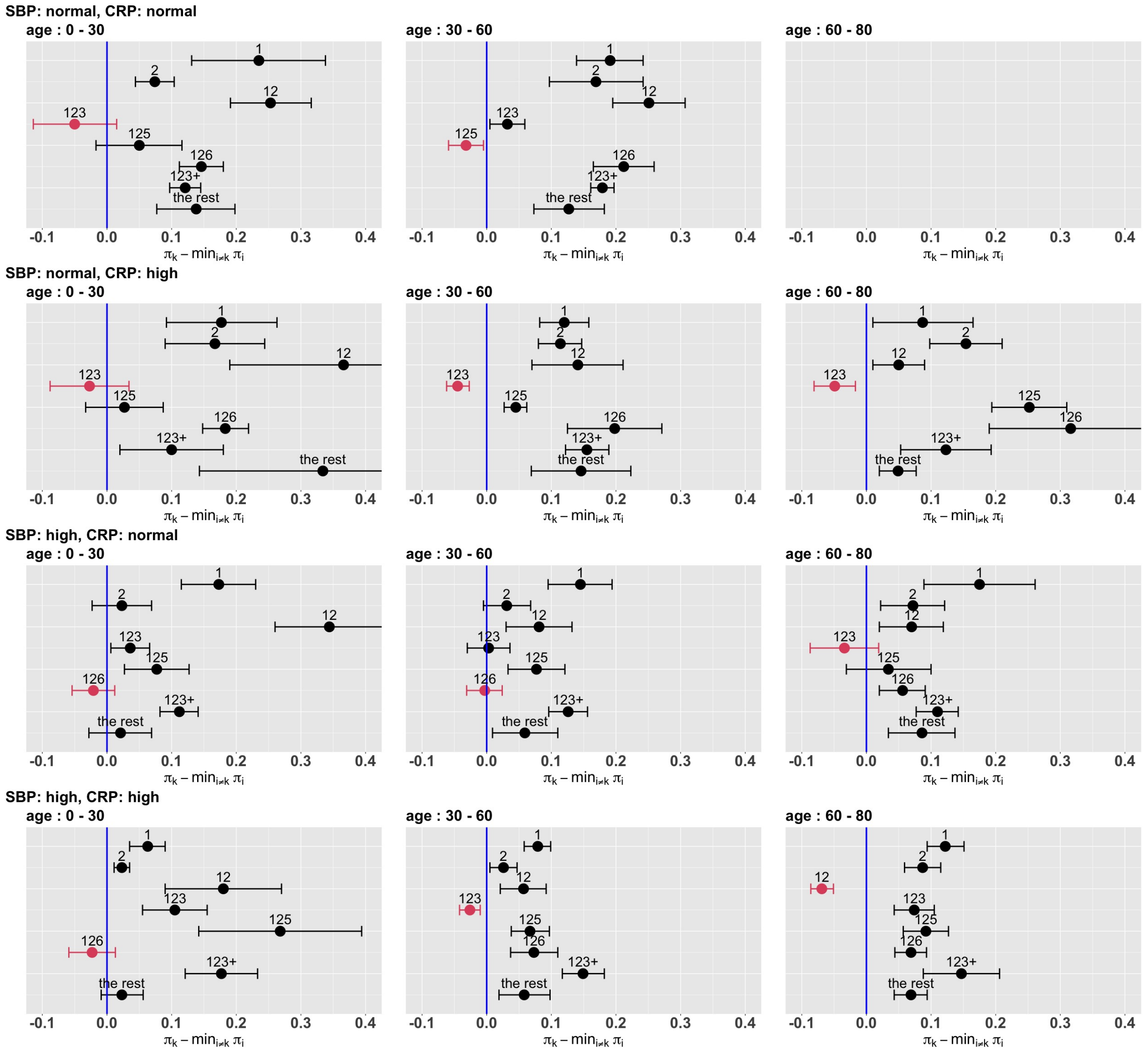

Figure S5: MCB sample best test (red) within each one of the $3 \times 2 \times 2$ subgroups defined jointly by age, SBP, and CRP.

Supplementary Tables

TABLE S1
Cross-validated negative log-likelihood of the chosen VMM method with different choices of `ntree`*.*

ntree	100	500	1000	2000
mean	0.361	0.362	0.361	0.361
sd	0.033	0.030	0.030	0.029
